# Roadside sobriety tests and attitudes toward a regulated cannabis market

**DOI:** 10.1186/1477-7517-4-4

**Published:** 2007-01-31

**Authors:** Alison Looby, Mitch Earleywine, Dale Gieringer

**Affiliations:** 1 University at Albany, State University of New York, 1400 Washington Ave, Department of Psychology, SS 369, Albany, New York, 12222, USA; 2 California NORML 2215-R Market St. #278, San Francisco CA 94114, USA

## Abstract

**Background:**

Many argue that prohibition creates more troubles than alternative policies, but fewer than half of American voters support a taxed and regulated market for cannabis. Some oppose a regulated market because of concerns about driving after smoking cannabis. Although a roadside sobriety test for impairment exists, few voters know about it. The widespread use of a roadside sobriety test that could detect recent cannabis use might lead some voters who currently oppose a regulated market to support it. In contrast, a question that primes respondents about the potential for driving after cannabis use might lead respondents to be less likely to support a regulated market.

**Methods:**

Phone interviews with a national sample of 1002 registered voters asked about support for a regulated cannabis market and support for such a market if a reliable roadside sobriety test were widely available.

**Results:**

In this sample of registered voters, 36% supported a regulated cannabis market. Exploratory chi-square tests revealed significantly higher support among men and Caucasians but no link to age or education. These demographic variables covaried significantly. Logistic regression revealed that gender, ethnicity, and political party were significant when all predictors were included. Support increased significantly with a reliable roadside sobriety test to 44%, but some respondents who had agreed to the regulated market no longer agreed when the sobriety test was mentioned. Logistic regression revealed that ethnicity and political affiliation were again significant predictors of support with a reliable sobriety test, but gender was no longer significant. None of these demographic variables could identify who would change their votes in response to the reliable roadside test.

**Conclusion:**

Increased awareness and use of roadside sobriety tests that detect recent cannabis use could increase support for a regulated cannabis market. Identifying concerns of voters who are not Caucasian or Democrats could help alter cannabis policy.

## Background

Despite frequent arguments that cannabis prohibition creates more problems than alternative policies, support for a taxed and regulated market has not reached a majority in the United States. Federal regulations on cannabis possession in America go back almost 70 years. Attitudes about these regulations have changed over this period, with different proportions of voters supporting prohibition in different eras [1]. Arrests for cannabis possession have increased dramatically since 1990, but cannabis prices have dropped while potency and rates of use among high school seniors has increased. At least a dozen individual states have passed laws permitting cannabis possession for medical use. Individual cities have passed legislation designed to make enforcement of prohibitions against cannabis possession a low priority for law enforcement. Some states have voted on legislation designed to alter laws prohibiting adult possession, too. Several authors have suggested that alternatives to prohibition would likely save considerable money as well as meaningful time for law enforcement officers and the court system [2,3]. Nevertheless, resistance to some plans for altered cannabis legislation remains.

Some supporters of cannabis prohibition show concern about driving a car after cannabis consumption. Many drugs impair driving, including over-the-counter antihistamines [4], as well as prescription anxiolytics and opiates [5]. Laboratory studies that administer cannabis to drivers have produced mixed results. Driving impairments are relatively minor at doses of 250 μg/kg or less, but increase at higher doses or with the addition of alcohol [6].

Supporters of cannabis prohibition might consider alternative cannabis regulations if law enforcement officers had a reliable index of cannabis intoxication in drivers. Physiological tests for cannabis or its metabolites often lag the intoxication experience so dramatically that a positive test would be a poor index of impairment. In contrast, field sobriety tests, which require behavioral observation of drivers performing various motor tasks, have the potential to identify currently impaired drivers. These tests would work well for impairment stemming from any drug. They could also detect drivers who might have problems related to fatigue or illness. It is unclear, however, if widespread use of such a test would alter attitudes about cannabis regulations.

Standard field sobriety tests usually include assessments of eye-tracking, walking a straight line, and standing on one leg [7]. The eye-tracking test requires following a moving object horizontally and then vertically with the eyes. People tend to have eye movements that are not smooth after using cannabis. Some also have problems holding their heads still during the task. These head movements appear particularly sensitive to cannabis intoxication. The walking task requires nine heel-to-toe steps along a straight line, turning, and repeating nine heal-to-toe steps in the opposite direction. After smoking cannabis, people are more likely to take the incorrect number of steps, fail to step heel-to-toe, require using their arms to maintain their balance, or stop walking in the middle of the test. The test for standing on one leg requires stretching one foot out in front of the body and counting aloud for 30 seconds starting from one-thousand. Intoxicated individuals are more likely to sway, put the extended foot down, or require arm movements to maintain balance. Standard field sobriety tests are moderate predictors of impairment caused by high and low doses of cannabis, though correct classification is enhanced as the dose increases. The application of field sobriety tests to individuals who have consumed cannabis in combination with other substances of abuse remains largely untested. Thus, although these tests provide a sensitive measure of impairment, it is unknown how they will fare when assessing drivers who are under the influence of a combination of drugs.

The current study asked a national sample of over 1,000 registered voters if they would support a taxed and regulated cannabis market comparable to the markets for alcohol, cigarettes, and gambling. These registered voters then responded to the same question but with the additional caveat about their support for a regulated market if a roadside sobriety test were available.

## Methods

### Overview

Zogby International conducted a telephone survey from August 22 through August 25, 2006. The survey included 53 questions on various topics. Phone numbers of registered voters were sampled with probabilities weighted by population size within area codes. Up to six calls were made to reach each sampled phone number.

### Survey questions

Participants provided demographic information, including gender, age, education, political party, income, and ethnicity. They also answered these questions:

1. Do you agree or disagree that marijuana should be legally taxed and regulated like liquor, tobacco and gambling? (Regulated market).

2. If police had a roadside impairment test for marijuana like the one they use for alcohol, would you support or oppose marijuana being legally taxed and regulated like liquor, tobacco and gambling? (Driving).

Responses to the question on a regulated market included 'agree', 'disagree', or 'not sure'. For the question that included the roadside impairment, responses included 'support', 'oppose', or 'not sure'.

### Data analyses

Because published data on attitudes about a taxed and regulated cannabis market in America are extremely sparse, we began with simple bivariate analyses. Our focus concerned the number of potential voters who supported the regulated market, so responses to each question were recoded into a dichotomous variable of 'support' or 'other'. We then examined links with each of the demographic variables. Because published data on this topic are rare, we chose alpha levels of .05 for each test, with the understanding that the Type I error rate for the entire set of questions would be higher. We report exact p-values (two-tailed) to aid interpretation.

Given the covariation among these demographic predictors, an assessment of their simultaneous effects appeared warranted. We simultaneously regressed the predictors from the initial analyses onto the binary outcome for each question in an effort to identify those that reliably predicted support for a regulated market. We also sought to identify which participants changed to or from an agree response when the roadside impairment test was mentioned in the question.

## Results

### Participant characteristics

The 1002 participants were registered voters and considered themselves somewhat or very likely to vote in the next election. The sample was 48% men and 77% Caucasian. Ages ranged from 18–95, with an average of 47.6 years (*SD *= 16.2). Education ranged from less than a high school diploma to college degree or more, with the largest group (48%) in the highest category. Political parties included Democrats (37%), Republicans (37%) and Independents (26%). Table 1 displays the correlations among these variables.

**Table 1:** Intercorrelations Among Predictors of Agreement to a Regulated Cannabis Market

Measure	1	2	3	4	5
1. Age	-				
2. Gender	-.019	-			
3. Education	-.107**	-.068*	-		
4. Race	.122**	.027	.007	-	
5. Political Party	.104**	.075*	-.070*	-.287**	-

Race is coded "Caucasian" or "other". Political party is coded "Democrat" or "other".

* *p *< .05 ** *p *< .01

### Bivariate analyses

#### Regulated market

Overall, 36% of the sample agreed that cannabis should be taxed and regulated when asked the first question. Point-biserial correlations revealed no significant link to age (*r *= -.02, *p *= .62) or education group (*r *= .01, *p *= .79). Exploratory chi-square tests revealed no differences among African-American, Asian, or Latino respondents (all *p*s > .10), but support was more likely among Caucasians (χ^2 ^= 4.19, Fischer's exact *p *= .043), and men (χ^2 ^= 4.28, Fischer's exact *p *= .041). No significant link was found between political party affiliation and support for a regulated market in the bivariate test (χ^2 ^= 3.37, Fischer's exact *p *= .076).

#### Driving

Overall, 44% of the sample agreed that cannabis should be taxed and regulated if a field sobriety test were widely available. The increase of 8% of the sample, however, was not uniformly from those who had disagreed with the regulated market statement. Those who agreed to the initial question on a regulated market were likely to agree with the driving statement (χ^2 ^= 347.40, *p *< .001). Nevertheless, 137 participants (13.9%) who had disagreed with the regulated market statement agreed with the driving statement. In contrast, 61 participants (6.1%) who had agreed with the regulated market statement disagreed with the driving statement. Point-biserial correlations revealed no significant link to age (*r *= -.03, *p *= .34) or education group (*r *= -.01, *p *= .75). Exploratory chi-square tests revealed that support was more likely among Caucasians (χ^2 ^= 5.86, Fischer's exact *p *= .016) and Democrats (χ^2 ^= 4.85, Fischer's exact *p *= .030), but showed no significant variation among other ethnic groups or political affiliations (all *p*s > .10). Gender was no longer significant (χ^2 ^= 1.72, Fischer's exact *p *= .203). Support from men increased from 39 to 46%; for women, support increased from 33 to 42%.

#### Change related to field sobriety test

McNemar's test for correlated proportions revealed that the percentage of people who agreed to the regulated market (36%) increased significantly with the addition of the mention of the field sobriety test (44%; χ^2 ^= 5.32, *p *< .05).

### Logistic regression

Logistic regressions were used for each question to predict whether a participant was likely to agree to a regulated cannabis market as a function of gender, age, education, Democratic party affiliation, and being Caucasian. Although political correctness would suggest that the analytic strategy of comparing Democrats to all other political parties and Caucasians to all other ethnic groups is unwise, the lack of variance among other political parties and ethnic groups revealed in the chi-square analyses suggested that dichotomizing these predictors along these lines would make for simpler interpretation of results. Although these results are somewhat redundant with the chi-square analyses above, the covariation among predictors suggested that an examination of all of them would help identify which ones accounted for unique variance in support for a regulated market.

For both regressions, there was evidence for multivariate normality, since Cook's distance for all cases fell within the acceptable range (between 0 and 1). Additionally, the Box-Tidwell approach was used to determine that there were no problems with linearity in the logit (Regulated Market: Wald statistic = 3.50, *p *> .05; Driving: Wald statistic = 3.33, *p *> .05). There is no evidence of multicollinearity since no bivariate correlation is greater than .90.

#### Regulated market

A test of the full model with all five predictors against a constant-only model was statistically significant, χ^2 ^= 18.01, *p *< .01, indicating that the predictors, as a set, reliably distinguished between the agreeing and disagreeing to a regulated cannabis market. The model was better at predicting those who disagreed than those who agreed, with 97.8% and 4% correctly predicted, respectively. The overall success rate of classification for the model was 63.6%.

Table 2 shows the unstandardized regression coefficients, Wald statistics, and odds ratios for each of the five predictors. According to the Wald criterion, gender, whether the participant was a Democrat, and whether the participant was Caucasian, reliably predicted whether one agreed to a regulated cannabis market. Examination of the odds ratios illustrates that females are 27% less likely than males to agree to a regulated market. Additionally, Democrats are 56% more likely than non-Democrats, and Caucasians are 64% more likely than participants of other ethnicities to agree.

**Table 2:** Logistic Regression Analysis of Support for a Regulated Cannabis Market (N = 1002)

Variables	*B*	Wald *χ*^2^-test	Odds Ratio
Gender	-0.31	5.31*	0.73
Age	-0.01	1.39	0.99
Education	0.03	0.11	1.03
Political Party	0.45	9.32**	1.56
Race	0.49	8.29**	1.64

Gender indicates the gender of the subject; 1 = male, 2 = female. Political party indicates whether one is a Democrat or not; 0 = party other than Democrat, 1 = Democrat. Race indicates whether one is Caucasian or not; 0 = race other than Caucasian, 1 = Caucasian.

* *p *< .05 ** *p *< .01

#### Driving

A test of the full model with all five predictors against a constant-only model was statistically significant, χ^2 ^= 21.92, *p *< .01. This model was still better at predicting those who disagreed (82.9%) than those who agreed (28.1%) to a regulated market. The decrease in the model's ability to predict those who would not support a regulated market resulted in the overall decrease in correct prediction for the model (58.7%); however, this model was more than 6 times better than the previous model in predicting those participants who would agree.

Table 3 shows the unstandardized regression coefficients, Wald statistics, and odds ratios for each of the five predictors. According to the Wald criterion, whether the participant was a Democrat and whether the participant was Caucasian reliably predicted whether one agreed to a regulated cannabis market. Gender was no longer a significant predictor. In this model, Democrats and Caucasians were even more likely than in the first model to support a regulated market, with Democrats being 65% more likely than non-Democrats and Caucasians being 78% more likely than participants of other ethnicities.

**Table 3:** Logistic Regression Analysis of Support for a Regulated Cannabis Market Including Roadside Impairment Tests (N = 1002)

Variables	*B*	Wald *χ*^2^-test	Odds Ratio
Gender	-0.24	3.43	0.79
Age	-0.01	3.19	0.99
Education	-0.01	0.03	0.99
Political Party	0.50	12.11*	1.65
Race	0.58	12.09*	1.78

Gender indicates the gender of the subject; 1 = male, 2 = female. Political party indicates whether one is a Democrat or not; 0 = party other than Democrat, 1 = Democrat. Race indicates whether one is Caucasian or not; 0 = race other than Caucasian, 1 = Caucasian.

* *p *< .01

#### Predicting change

Two additional logistic regressions were run to predict which participants would alter their opinion regarding a regulated cannabis market after learning of a roadside impairment test. Again, gender, age, education, Democratic-party affiliation, and being Caucasian were used as the predictor variables to determine which subjects would be most likely to disagree to the first question but agree to the second, and vice versa. Neither overall model was significant [changed from agree to disagree: χ^2 ^= 3.74, *p *= .59; changed from disagree to agree: χ^2 ^= 4.09, *p *= .54]. Thus, there were no significant predictors to account for those participants who changed their stance on a regulated cannabis market.

## Discussion

We sought to determine rates of support for a regulated cannabis market that would be similar to the markets for alcohol, cigarettes, and gambling. We also examined if the widespread use of a roadside sobriety test that could detect driving after cannabis consumption could increase support for a regulated market. Approximately one-third of the participants supported a regulated market when asked directly. In addition, over 40% of the participants supported a regulated market once the caveat of including roadside impairment tests for cannabis was addressed. We then examined a range of demographic variables in an attempt to predict who would support a regulated cannabis market. Ethnicity, gender, and political affiliation accounted for significant variance. Men, Democrats, and Caucasians were more likely to support a regulated market. Once the idea of roadside impairment tests was addressed, Caucasian and Democrat participants were even more likely to support a regulated market.

Though support for a regulated cannabis market increased with the discussion of roadside impairment tests, some of the participants who had originally agreed with this proposal no longer supported it. It is difficult to determine the reason for this finding, but perhaps priming the idea of cannabis-related driving problems led participants who had not considered this issue when first asked about a taxed and regulated market to no longer support such a market. Possibly these respondents had not considered issues related to driving when they answered the first question, and the mention of driving led them to change their minds. Additionally, we were unable to predict which participants would change their stance on the issue following the second question.

This sample has many strengths related to the focus on registered voters and the large sample. One limitation of this study is the use of the telephone survey. Due to the sensitive topic of drug legalization, many respondents may not have wished to state their true opinion over the phone. Several researchers have established that response bias is present in telephone interviews [8,9]. This finding is particularly salient in African American populations, possibly due to distrust of the research process or guardedness about confidentiality assurances [10,11]. Furthermore, social desirability may be another factor contributing to bias in telephone interviews [8]. Nevertheless, Meyer, Rossano, Ellis, and Bradford [12] and Ellen et al. [13] claim that accurate results can be obtained through telephone interviews, even when inquiring about sensitive topics. Further research on this topic should use alternative interview techniques to control for bias resulting from the use of a telephone survey.

In conclusion, support for a regulated cannabis market has been obtained from a variety of participants from around the country. This level of support increased with the additional provision of roadside impairment tests, particularly among Caucasians and Democrats. Campaigns that educate registered voters about the existence of roadside impairment tests for cannabis have the potential to increase support for reform. With the widespread use of a roadside impairment tests, many voters may alter their perceptions regarding the legalization and regulation of cannabis. These data also suggest that a better understanding of the concerns of voters who are not Caucasian or Democrats might enhance support for changes in cannabis policy.

## Competing interests

The authors have no financial competing interests to report. ME and DG both work for organizations devoted to altering cannabis policy.

## Authors' contributions

AL conducted analyses and drafted the manuscript. ME assisted in drafting the manuscript, conducted analyses, and assisted in designing the study. DG assisted in designing the study and in data collection. All authors read and approved the final manuscript.
